# Additional Plate Fixation of Hinge Fractures After Varisation Distal Femoral Osteotomies Provides Favorable Torsional Stability: A Biomechanical Study

**DOI:** 10.1177/03635465231206947

**Published:** 2023-11-07

**Authors:** Christian Peez, Arian Grosse-Allermann, Adrian Deichsel, Michael J. Raschke, Johannes Glasbrenner, Thorben Briese, Jens Wermers, Elmar Herbst, Christoph Kittl

**Affiliations:** * Department of Trauma, Hand and Reconstructive Surgery, University Hospital Münster, Münster, Germany; Investigation performed at the Department of Trauma, Hand and Reconstructive Surgery, University Hospital Münster, Münster, Germany

**Keywords:** biomechanics, hinge fixation, hinge fracture, torsional instability, varisation distal femur osteotomy

## Abstract

**Background:**

Hinge fractures are considered risk factors for delayed or nonunion of the osteotomy gap in distal femoral osteotomies (DFOs). Limited evidence exists regarding the treatment of hinge fractures after DFO, which could improve stability and thus bone healing.

**Purpose:**

To (1) examine the effect of hinge fractures on the biomechanical properties of the bone-implant construct, (2) evaluate the biomechanical advantages of an additional fixation of a hinge fracture, and (3) test the biomechanical properties of different types of varisation DFOs.

**Study Design:**

Controlled laboratory study.

**Methods:**

A total of 32 fresh-frozen human distal femora equally underwent medial closing wedge DFO or lateral opening wedge DFO using a unilateral locking compression plate. The following conditions were serially tested: (1) preserved hinge; (2) hinge fracture along the osteotomy plane; (3) screw fixation of the hinge fracture; and (4) locking T-plate fixation of the hinge fracture. Using a servo-hydraulic materials testing machine, we subjected each construct to 15 cycles of axial compression (400 N; 20 N/s) and internal and external rotational loads (10 N·m; 0.5 N·m/s) to evaluate the stiffness. The axial and torsional hinge displacement was recorded using a 3-dimensional optical measuring system. Repeated-measures 1-way analysis of variance and post hoc Bonferroni correction were used for multiple comparisons. Statistical significance was set at *P* < .05.

**Results:**

Independent from the type of osteotomy, a fractured hinge significantly (*P* < .001) increased rotational displacement and reduced stiffness of the bone-implant construct, resulting in ≥1.92 mm increased displacement and ≥70% reduced stiffness in each rotational direction, while the axial stiffness remained unchanged. For both procedures, neither a screw nor a plate could restore intact rotational stiffness (*P* < .01), while only the plate was able to restore intact rotational displacement. However, the plate always performed better compared with the screw, with significantly higher and lower values for stiffness (+38% to +53%; *P* < .05) and displacement (–55% to −72%; *P* < .01), respectively, in ≥1 rotational direction. At the same time, the type of osteotomy did not significantly affect axial and torsional stability.

**Conclusion:**

Hinge fractures after medial closing wedge DFO and lateral opening wedge DFO caused decreased bone-implant construct rotational stiffness and increased fracture-site displacement. In contrast, the axial stiffness remained unchanged in the cadaveric model.

**Clinical Relevance:**

When considering an osteosynthesis of a hinge fracture in a DFO, an additional plate fixation was the construct with the highest stiffness and least displacement, which could restore intact hinge rotational displacement.

Varisation distal femoral osteotomy (DFO) is a well-established technique to treat valgus deformity with mild to moderate lateral compartment osteoarthritis,^5,7,10,32,33,36^ patellofemoral instability,^9,15,22,28,34^ lateral meniscal deficiency, or in combination with cartilage regenerative procedures.^5^ The knee joint can be realigned by either a medial closing wedge (MCW)^41^ or a lateral opening wedge (LOW)^12^ technique. Excellent clinical, functional, and radiographic outcomes and survivorships of almost 80% at 10 years have been shown for both procedures.^5,10,11,13,21,28,33^ The associated high rates of reoperation, hardware failure, and loss of correction could be reduced by establishing angular stable plate systems,^6,18,23,24,40^ while malunion of the osteotomy gap and hinge fractures remain the major concern of both techniques.^6,8,40^

These fractures at the hinge of the osteotomy site are frequently observed complications with a reported incidence on plain radiography of up to 35% after MCW-DFO^28^ and 48% after LOW-DFO.^31^ They can be even higher (60%) when measured using computed tomography.^29^ The resulting instability and reduced stiffness of the bone-implant construct^3,25,30^ may be one reason for delayed consolidation, as evidenced by the increased risk of malunions with associated rates of up to 6.7% and 7.3% after hinge fractures in MCW-DFO^26^ and LOW-DFO,^23,24^ respectively. Thus, several strategies have been reported to prevent these fractures, like the protective hinge wire^35^ or positioning of the medial hinge distal to the adductor tubercle.^39^ However, a certain rate of hinge fractures remains,^35,39^ which may compromise functional outcomes and patient-reported outcome measures.^16,27^

Thus, this study aimed to (1) examine the effect of hinge fractures on the biomechanical properties of the bone-implant construct, (2) evaluate the biomechanical advantages of an additional fixation of a hinge fracture, and (3) test the biomechanical properties of the different types of varisation DFOs (MCW vs LOW and monoplanar vs biplanar). First, it was hypothesized that hinge fractures would reduce stiffness and increase interfragmentary instability across the osteotomy gap. Second, an additional fixation of a hinge fracture would restore stiffness and interfragmentary instability. Finally, a biplanar osteotomy and an MCW-DFO would be more stable (increased stiffness and reduced instability) compared with a monopolar osteotomy and a LOW-DFO, respectively.

## Methods

A total of 32 fresh-frozen human cadaveric femora, with a mean age of 79.1 ± 5.6 years (12 women, 20 men), from a local tissue bank were used for biomechanical testing. The knee specimens were dissected and tested biomechanically under permission of the Law on Corpses, Burials, and Cemeteries (Burial Law) of the state of Schleswig-Holstein of February 4, 2005, section II, § 9 (Anatomical Opening of Corpses).

### Specimen Preparation and Surgical Techniques

The femora were stored at −20° C and thawed for 24 hours at room temperature before testing. Based on sex and age, the specimens were equally assigned to 2 clusters according to the technique for realignment surgery: MCW-DFO and LOW-DFO. Each osteotomy technique was performed as a monoplanar and biplanar osteotomy in 8 femora: monoplanar MCW-DFO, biplanar MCW-DFO, monoplanar LOW-DFO, and biplanar LOW-DFO.

**Figure 1.**
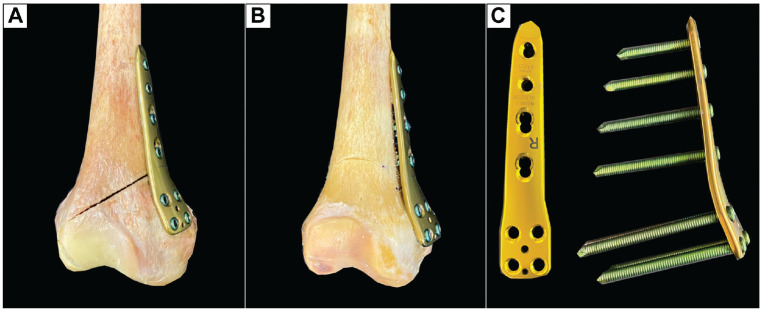
A right distal femur after MCW-DFO. (A) Monoplanar MCW-DFO. (B) Biplanar MCW-DFO. (C) Locking compression plate system for the medial distal femur. MCW-DFO, medial closing wedge–varisation distal femur osteotomy.

The MCW-DFO was performed using the technique described by Wylie and Maak^41^ with minor modifications. Using an aiming guide (KARL STORZ), 2 parallel 2.4-mm K-wires were drilled through the distal femoral metaphysis starting 10 mm proximal to the medial epicondyle, placing the tips of the K-wires on the upper border of the lateral femoral epicondyle, which marked the hinge position in the safe zone.^20^ In previous clinical studies, a mean varus correction of 8° has been reported for MCW osteotomies.^1,33^ Thus, a 7-mm wedge corresponding to an 8° varus correction^41^ was formed by placing a further two 2.4-mm K-wires proximally to ensure comparability with the aforementioned studies.^1,33^ For biplanar MCW-DFO (Figure 1B), a frontal osteotomy was first performed in the anterior quarter of the distal femoral metaphysis at an angle of 100° to the planned correction level, which was followed by an axial osteotomy along the K-wires using an oscillating saw. For the monoplanar MCW-DFO, only the axial osteotomy was performed (Figure 1A). After a 10 mm–wide lateral hinge was created, the medial bone wedge was removed, and the osteotomy gap was closed while preserving an intact lateral hinge. Then, the MCW-DFO was fixed using a medial locking compression plate (LCP) system (4.5- to 5.0-mm LCP; TomoFix medial distal femur; DePuy Synthes; Johnson & Johnson Medical Device Company) (Figure 1C). For this, 4 unicortical locking screws were placed in the metaphyseal segment. After compressing the osteotomy site using reduction forceps, we fixed the diaphyseal segment with 4 bicortical locking screws. If the most distal screw of the diaphyseal segment crossed the osteotomy plane, it was removed to ensure a nonintersected osteotomy gap.

**Figure 2.**
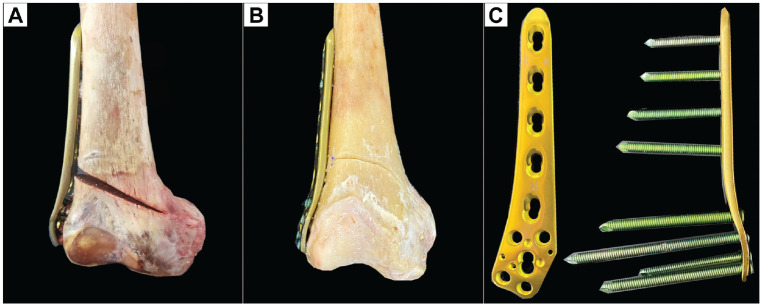
A right distal femur after LOW-DFO. (A) Monoplanar LOW-DFO. (B) Biplanar LOW-DFO. (C) Locking compression plate system for the lateral distal femur. LOW-DFO, lateral opening wedge–varisation distal femur osteotomy.

The LOW-DFO was performed using the technique described by Feucht et al,^12^ with minor modifications. Using an aiming guide, we drilled 2 parallel 2.4-mm K-wires through the distal femoral metaphysis starting 30 mm proximal to the lateral epicondyle, placing the tips of the K-wires distal to the adductor tubercle and 10 mm lateral to the medial epicondyle, which marked the ideal hinge position.^39^ For biplanar LOW-DFO (Figure 2B), a frontal osteotomy was first performed in the anterior quarter of the distal femoral metaphysis at an angle of 100° to the planned correction level, followed by the axial osteotomy along the K-wires using an oscillating saw, whereas only the axial osteotomy was performed for the monoplanar LOW-DFO (Figure 2A). After preserving the medial cortex and consistent with previous studies, we performed a lateral open wedge with a height of 10 mm.^5,10,11,21,36^ Then, the LOW-DFO was fixed using a lateral LCP system (4.5-mm to 5.0-mm LCP; TomoFix lateral distal femur; DePuy Synthes; Johnson & Johnson Medical Device Company) (Figure 2C). Five unicortical locking screws were placed in the metaphyseal segment, whereas the diaphyseal segment was fixed with 4 bicortical locking screws. If the most distal screw of the diaphyseal segment crossed the osteotomy plane, it was removed to ensure a nonintersected osteotomy gap.

### Testing Conditions

**Figure 3.**
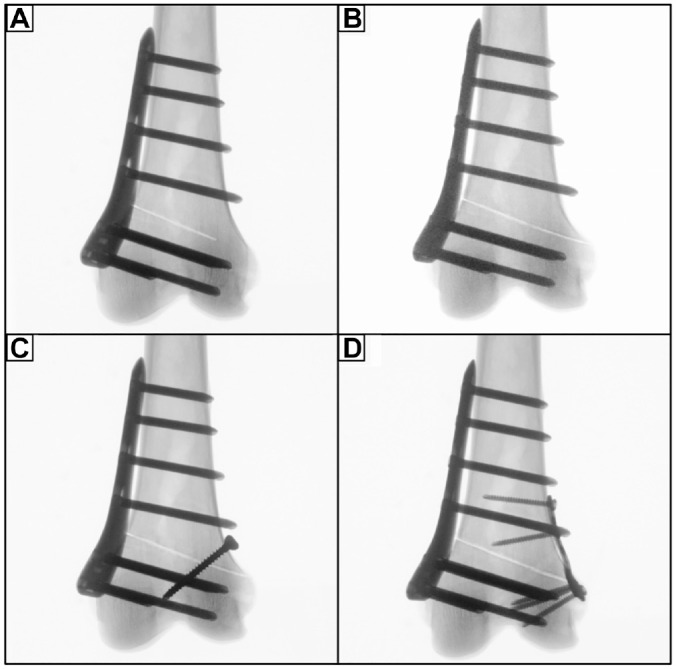
Radiographs of a left distal femur after MCW-DFO, which was fixed by an ipsilateral locking compression plate system. (A) Preserved lateral hinge (intact). (B) Construct with a lateral hinge fracture in the direction of the osteotomy plane (fracture). (C) Additional lag screw fixation of the lateral hinge fracture (screw). (D) Additional T-plate fixation of the lateral hinge fracture (plate). MCW-DFO, medial closing wedge–varisation distal femur osteotomy.

**Figure 4.**
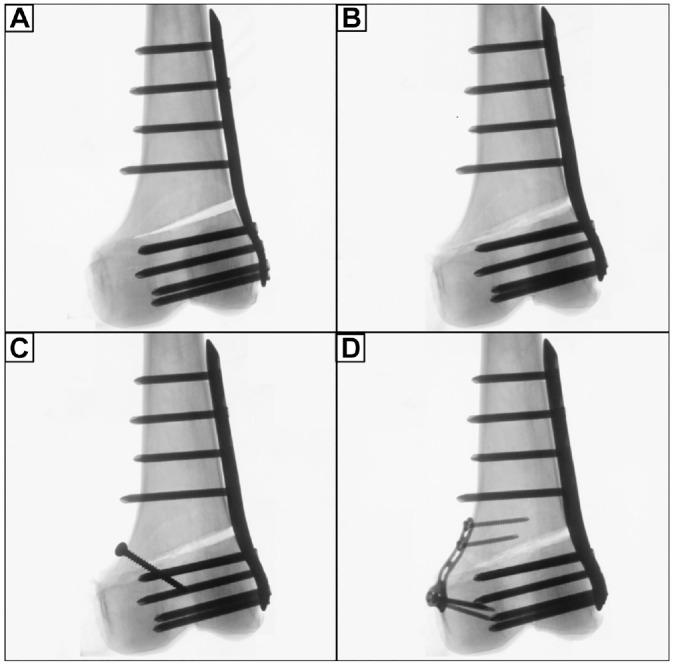
Radiographs of a left distal femur after LOW-DFO, which was fixed by an ipsilateral locking compression plate system. (A) Preserved medial hinge (intact). (B) Construct with a medial hinge fracture in the direction of the osteotomy plane (fracture). (C) Additional lag screw fixation of the medial hinge fracture (screw). (D) Additional T-plate fixation of the medial hinge fracture (plate). LCW-DFO, lateral closing wedge–varisation distal femur osteotomy.

The following conditions were serially tested: (1) unilateral locking plate fixation with a preserved hinge (intact); (2) unilateral locking plate fixation combined with hinge fracture (fracture); (3) unilateral locking plate fixation combined with additional lag screw fixation of the hinge fracture (screw); and (4) unilateral locking plate fixation combined with additional locking T-plate fixation of the hinge fracture (plate) (Figures 3 and 4).

For the fracture, a type 1 hinge fracture type, according to Winkler et al,^39^ was simulated by osteotomizing the hinge along the osteotomy plane. This fracture morphology represents the most common hinge fracture type after MCW-DFO^29^ and LOW-DFO,^27,39^ for which an increased risk of delayed union of the osteotomy gap has been shown, regardless of the osteotomy technique used.^16,27^ A complete lack of the corresponding cortex was ensured using an oscillating saw. The idea was to imitate a state of maximum instability by creating an osteotomy instead of a simple fracture on the opposite cortex.

For the screw, a 44 × 4.5–mm cortical screw (DePuy Synthes; Johnson & Johnson Medical Device Company) was used as a lag screw. To guarantee maximum compression, the screw was inserted from 1.5 to 2 mm proximal to the fracture line into the direction crossing the osteotomy perpendicularly. For the plate, an additional 3.5-mm locking T-plate was used (3.5 mm T-LCP; DePuy Synthes; Johnson & Johnson Medical Device Company). The T-shaped design of the LCP was used to ensure maximum restraint against rotational forces.

### Biomechanical Testing

Mechanical testing was performed using a servo-hydraulic materials testing machine (Model 8874; Instron GmbH). The accuracy of the loading cell was ±.005%, allowing a position control with an accuracy of ±0.5% for the testing unit. The distal end of each femur was cemented in a custom-designed vise fixture using polymethylmethacrylate for unconstrained positioning of the specimens before testing. Then, the specimens were positioned in a way that the mechanical load axis of the femur ran parallel to the test actuator axis, which allowed axial testing with simulation of physiological loads on the osteotomy gap during the bipedal stance phase. The proximal end of the specimen was fixed in a clamp, which was connected directly to the loading cell.

According to previous studies,^2,3,25^ a nondestructible, quasi-static cyclic loading test was conducted using 15 cycles of axial compression (400 N; 20 N/s) and torsional load in internal rotation (IR) and external rotation (ER) (10 N·m in IR/ER; 0.5 N·m/s). For all test conditions, standard force-displacement curves were then generated.

### Motion Tracking

**Figure 5.**
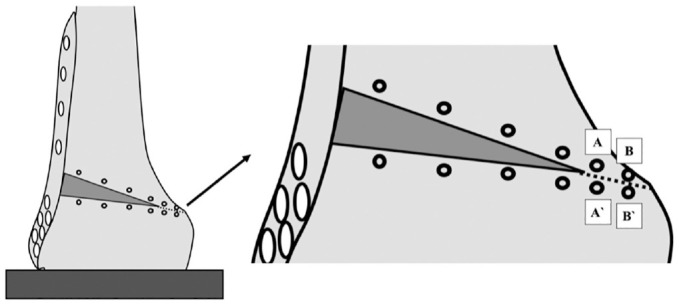
Schematic illustration of a monoplanar lateral opening wedge distal femoral osteotomy during motion tracking analysis. The optical markers (black eyelets) with a diameter of 3 mm were evenly placed on the anterior cortex of the distal femur along the proximal and distal edge of the osteotomy plane. The mean differences between all corresponding proximal and distal markers were analyzed for axial displacement. Four optical markers were attached to the femur 5 mm from the hinge line at the hinge site. Optical markers *A* and *B* were placed proximal, and optical markers *A′* and *B′* were placed distal to the hinge line. To quantify hinge rotational displacement under torsional loads, we recorded the relative shifts of marker *A* to *A′* and marker *B* to *B′* using an optical 3-dimensional measuring system.

For each construct, the interfragmentary displacement was detected under axial compression and torsional loads over the entire duration of cyclic loading testing using an optical 3-dimensional measuring system (GOM Aramis; GOM GmbH). Optical markers with a diameter of 3 mm were evenly placed on the anterior cortex of the distal femur along the proximal and distal edges of the osteotomy plane. At the hinge site, 4 optical markers were attached to the femur 5 mm from the hinge line (proximal and distal) so that hinge displacement could be dynamically recorded over the entire cyclic loading test (Figure 5).

### Data Acquisition

The axial stiffness and torsional stiffness in IR or ER of each construct—defined as the steepest slope of the force-displacement curve—were quantified from the last 3 loading cycle deformation curves to consider settling effects during testing.

The displacement was assessed using the metrology software (GOM Aramis; GOM GmbH) in an *x-y-z* femoral coordinate system at peak loading conditions of the last 3 loading cycles to consider settling effects during testing. To calculate axial displacement (*y*-axis), the mean differences between all corresponding proximal and distal markers were analyzed. For hinge rotational displacement, the mean differences of the hinge markers (*A* to *A′* and marker *B* to *B′* in Figure 5) were separately calculated in IR and ER (*z*-axis).

### Statistical Analysis

In a previous clinical study, a dislocation at the hinge site >2 mm could be considered a critical threshold value for promoting delayed bone union.^31^ For this, an a priori power analysis showed that a sample size of 6 specimens per group would lead to a 90% power to detect a difference of 2 mm between the means at the ß > 0.8 level.

Statistical analysis was performed using MATLAB (R2020a; MathWorks) and Prism Version 9 (GraphPad Software). The data normality was tested using the Shapiro-Wilk test. A repeated-measures 1-way analysis of variance was performed for each hypothesis. Post hoc testing with Bonferroni correction was used to control multiple comparisons. Significance was set at *P* < .05. The data were presented as mean and standard deviation.

## Results

### Effect of Hinge Fractures

**Figure 6.**
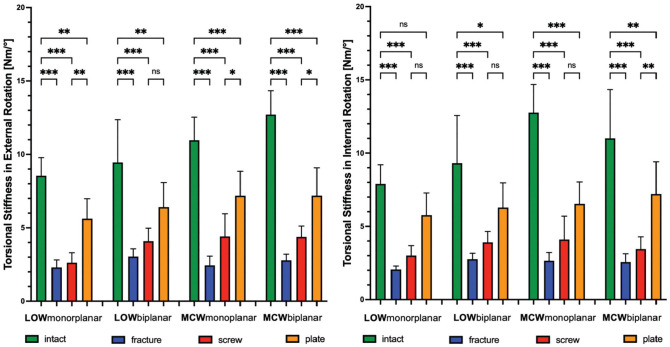
Changes (N·m/deg) in torsional stiffness after LOW-DFO and MCW-DFO. Error bars indicate mean and SD. DFO, distal femoral osteotomies; fracture, unilateral locking plate fixation combined with hinge fracture; intact, unilateral locking plate fixation with preserved hinge; LOW, lateral opening wedge; MCW, medial closing wedge; ns, not significant; plate, unilateral locking plate fixation combined with additional locking plate fixation of the hinge fracture; screw, unilateral locking plate fixation combined with additional lag screw fixation of the hinge fracture. *P < .05; **P <.01; ***P < .001.

**Figure 7.**
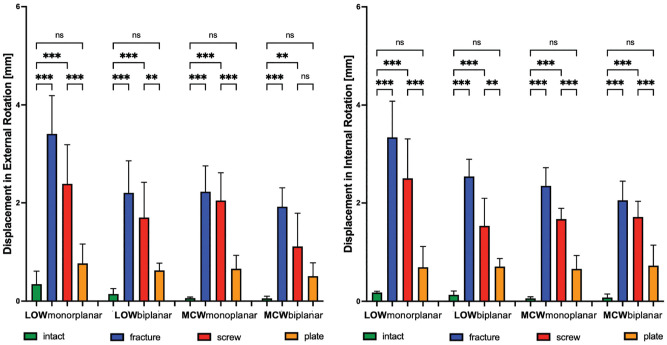
Changes (mm) in rotational displacement after LOW-DFO and MCW-DFO. Error bars indicate mean and SD. DFO, distal femoral osteotomies; fracture, unilateral locking plate fixation combined with hinge fracture; intact, unilateral locking plate fixation with preserved hinge; LOW, lateral opening wedge; MCW, medial closing wedge; ns, not significant; plate, unilateral locking plate fixation combined with additional locking plate fixation of the hinge fracture; screw, unilateral locking plate fixation combined with additional lag screw fixation of the hinge fracture. **P <.01; ***P < .001.

Independent from the type of osteotomy, a fractured hinge significantly (*P* < .001) increased rotational displacement and reduced stiffness of the bone-implant construct. This resulted in ≥3.3 mm increased displacement at the hinge site and a 73% reduced construct stiffness under rotational loads in a LOW monoplanar osteotomy. In an MCW biplanar osteotomy, there was ≥1.9 mm IR and ER displacement and ≥70% reduced torsional stiffness in each rotational direction, which was the lowest among the types of osteotomy (Figures 6 and 7).

**Figure 8.**
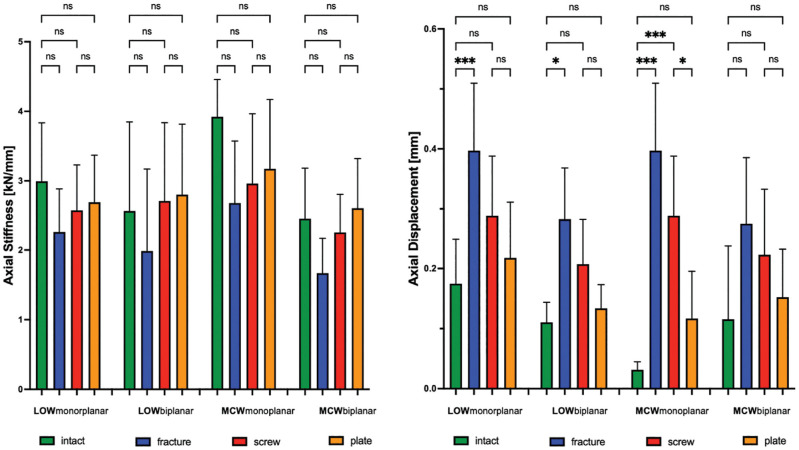
Changes of axial stiffness (kN/mm) and axial displacement (mm) after LOW-DFO and MCW-DFO. Error bars indicate mean and SD. DFO, distal femoral osteotomies; fracture, unilateral locking plate fixation combined with hinge fracture; intact, unilateral locking plate fixation with preserved hinge; LOW, lateral opening wedge; MCW, medial closing wedge; ns, not significant; plate, unilateral locking plate fixation combined with additional locking plate fixation of the hinge fracture; screw, unilateral locking plate fixation combined with additional lag screw fixation of the hinge fracture. *P < .05; ***P < .001.

When looking at axial load, a hinge fracture had no significant effect on the axial stiffness. However, the displacement after LOW-DFO (monoplanar, *P* <.001; biplanar, *P* < .05) and monoplanar MCW-DFO (*P* < .001) was increased compared with the intact state, while no significance was shown for the MCW biplanar osteotomy (Table 1 and 2, Figure 8).

**Table 1:** Axial Stiffness, Axial Displacement, Torsional Stiffness, and Displacement After Monoplanar and Biplanar LOW-DFO^*a*^

Parameters	Monoplanar LOW-DFO			
	Intact	Fracture	Screw	Plate
Axial stiffness, kN/mm (*P*)	2.99 ± 0.83	2.26 ± 0.62 (.972)	2.57 ± 0.65 (>.999)	2.69 ± 0.67 (>.999)
Axial displacement, mm (*P*)	0.17 ± 0.07	0.39 ± 0.11 **(.001)**	0.28 ± 0.09 (.519)	0.21 ± 0.09 (>.999)
Torsional stiffness ER, N·m/deg (*P*)	8.54 ± 1.23	2.30 ± 0.51 **(<.001)**	2.62 ± 0.67 **(<.001)**	5.62 ± 1.35 ** (.007)**
Torsional stiffness IR, N·m/deg (*P*)	7.89 ± 1.30	2.05 ± 0.23 **(<.001)**	3 ± 0.68 **(<.001)**	5.77 ± 1.51 (.518)
Displacement ER, mm (*P*)	0.34 ± 0.26	3.40 ± 0.77 **(<.001)**	2.39 ± 0.79 **(<.001)**	0.76 ± 0.39 (.964)
Displacement IR, mm (*P*)	0.17 ± 0.02	3.34 ± 0.73 **(<.001)**	2.50 ± 0.80 **(<.001)**	0.69 ± 0.42 (.509)
Parameters	Biplanar LOW-DFO			
	Intact	Fracture	Screw	Plate
Axial stiffness, kN/mm (*P*)	2.56 ± 1.28	1.98 ± 1.18 (.997)	2.71 ± 1.12 (>.999)	2.80 ± 1.01 (>.999)
Axial displacement, mm (*P*)	0.11 ± 0.03	0.28 ± 0.85 **(.021)**	0.20 ± 0.07 (.787)	0.13 ± 0.03 (>.999)
Torsional stiffness ER, N·m/deg (*P*)	9.45 ± 2.91	3.04 ± 0.52 **(<.001)**	4.08 ± 0.88 **(<.001)**	6.41 ± 1.66 ** (.004)**
Torsional stiffness IR, N·m/deg (*P*)	9.31 ± 3.24	2.75 ± 0.40 **(<.001)**	3.91 ± 0.74 **(<.001)**	6.28 ± 1.68 ** (.044)**
Displacement ER, mm (*P*)	0.14 ± 0.11	2.21 ± 0.65 **(<.001)**	1.70 ± 0.72 **(<.001)**	0.62 ± 0.14 (.891)
Displacement IR, mm (*P*)	0.13 ± 0.07	2.54 ± 0.35 **(<.001)**	1.53 ± 0.56 **(<.001)**	0.70 ± 0.16 (.294)

a Data are presented as mean ± SD. Bold values indicate significant *P* values. ER, external rotation; IR, internal rotation; LOW-DFO, lateral opening wedge distal femoral osteotomy.

**Table 2:** Axial Stiffness, Axial Displacement, Torsional Stiffness, and Displacement After Monoplanar and Biplanar MCW-DFO^*a*^

Parameters	Monoplanar MCW-DFO			
	Intact	Fracture	Screw	Plate
Axial stiffness, kN/mm (*P*)	3.92 ± 0.53	2.68 ± 0.89 (.308)	2.96 ± 1 (.762)	3.17 ± 0.99 (.964)
Axial displacement, mm (*P*)	0.03 ± 0.01	0.39 ± 0.11 **(<.001)**	0.28 ± 0.09 **(<.001)**	0.11 ± 0.07 (.913)
Torsional stiffness ER, N·m/deg (*P*)	12.77 ± 1.91	2.65 ± 0.55 **(<.001)**	4.41 ± 1.54 **(<.001)**	7.18 ± 1.66 **(<.001)**
Torsional stiffness IR, N·m/deg (*P*)	11.01 ± 3.32	2.44 ± 0.62 **(<.001)**	4.10 ± 1.59 **(<.001)**	6.53 ± 1.59 **(<.001)**
Displacement ER, mm (*P*)	0.06 ± 0.01	2.23 ± 0.52 **(<.001)**	2.04 ± 0.57 **(<.001)**	0.65 ± 0.27 (.603)
Displacement IR, mm (*P*)	0.06 ± 0.02	2.35 ± 0.37 **(<.001)**	1.67 ± 0.21 **(<.001)**	0.65 ± 0.27 (.237)
Parameters	Biplanar MCW-DFO			
	Intact	Fracture	Screw	Plate
Axial stiffness, kN/mm (*P*)	2.45 ± 0.72	1.61 ± 0.56 (.943)	2.25 ± 0.54 (>.999)	2.60 ± 0.71 (.999)
Axial displacement, mm (*P*)	0.07 ± 0.01	0.27 ± 0.11 (.052)	0.22 ± 0.10 (.631)	0.15 ± 0.08 (>.999)
Torsional stiffness ER, N·m/deg (*P*)	12.77 ± 1.91	2.78 ± 0.41 **(<.001)**	4.38 ± 0.73 **(<.001)**	7.20 ± 2.19 **(<.001)**
Torsional stiffness IR, N·m/deg (*P*)	10.46 ± 2.21	2.56 ± 0.56 **(<.001)**	3.45 ± 0.83 **(<.001)**	7.19 ± 1.89 **(.002)**
Displacement ER, mm (*P*)	.05 ± 0.04	1.92 ± 0.39 **(<.001)**	1.11 ± 0.67 **(.004)**	0.50 ± 0.27 (.936)
Displacement IR, mm (P)	0.07 ± 0.06	2.05 ± 0.39 **(<.001)**	1.71 ± 0.32 **(<.001)**	0.72 ± 0.54 (.129)

a Data are presented as mean ± SD. Bold values indicate significant *P* values. ER, external rotation; IR, internal rotation; MCW-DFO, medial closing wedge distal femoral osteotomy.

### Effect of Hinge Fixation

Regardless of the osteotomy technique, neither a screw nor a plate could restore intact rotational stiffness, and only the plate was able to restore rotational displacement. In ≥1 rotational direction, the plate always performed better compared with the screw, with significantly higher values for stiffness (+38% to +53%; *P* < .05) and lower values for displacement (–55% to −72%; *P* < .01) compared with the screw (Tables 1 and 2; Figures 6 and 7). When looking at axial load, the screw and the plate could restore intact stiffness and displacement, except for the axial displacement in the screw group after monoplanar MCW osteotomy (*P* < .001). Unlike rotational loads, there was no significant difference between the screw and the plate, except for the monoplanar MCW osteotomy, where the plate showed lower displacement compared with the screw (*P* <.05) (Tables 1 and 2; Figure 8).

### Effects of LOW or MCW Techniques

There was no uniform difference regarding stiffness and displacement between the LOW and MCW techniques (Tables 1 and 2).

### Effects of Monoplanar or Biplanar Osteotomy Techniques

There was no significant difference between the monoplanar or biplanar techniques regarding rotational stiffness. The biplanar osteotomy, however, showed significantly less displacement in IR and ER for the LOW technique with a fractured hinge and ER with a screw osteosynthesis of the hinge (*P* < .05) (Table 1). For the MCW technique, this was only significant for ER in the screw group (*P* < .01) (Table 2). Again, there was no difference in axial stiffness and displacement.

The summarized results after biomechanical testing of the different testing conditions are shown in Tables 1 and 2.

## Discussion

The most important finding of the present study was that hinge fractures after an MCW-DFO and an LOW-DFO caused significantly increased rotational displacement at the fracture site and reduced torsional stiffness of the bone-implant construct. Under axial loads, hinge fractures had no significant effect on the axial stiffness but caused significantly increased axial displacement. An additional plate fixation of hinge fractures was the construct with the highest stiffness and least displacement, which could restore intact hinge rotational displacement but was not able to restore rotational stiffness. Based on our third hypothesis, no clear difference between the osteotomy techniques could be found. However, there was a trend that the MCW and biplanar technique performed better, at least in some groups.

Although, from a biomechanical point of view, the destabilizing effect of hinge fractures remains the key concern in DFOs, only a few biomechanical studies have evaluated different strategies for additional hinge fixation. In 2015, Batista et al^3^ investigated the biomechanical properties of buttressing hinge fractures using a supplemental screw fixation. In a synthetic bone model, they compared a locking and angle blade plate fixation after a monoplanar LOW osteotomy with and without hinge fracture fixation. Similar to the present study, they found that an intact medial cortex provided the highest axial and torsional stiffness. A fractured hinge caused a significant decrease in axial (32.6% vs 24.5% in the present study) and torsional (25.6% vs ≥70% in the present study) stiffness, which were higher for the axial stiffness but lower for the torsional stiffness compared with the present study. These differences may come from the noncyclic higher axial (1500 N vs 400 N in the present study), lower rotational loads (7 N·m vs 10 N·m in the present study), and the 10-mm gap, which simulated the hinge fracture in their study. Additional screw fixation of the hinge fracture could thus restore rotational stiffness, whereas in the present study, none of the tested osteosyntheses could restore the intact stiffness. This again may come from the aforementioned differences and from a larger screw used in their study (6.5 mm vs 4.5 mm in the present study), as a larger screw diameter correlates with a higher biomechanical stability.^14^

A more recent study by Matsushita et al^25^ examined the biomechanical effects of an additional screw and plate fixation of hinge fractures after monoplanar MCW osteotomies in a cadaveric knee model. Similar to the present study, it was found that a fractured lateral hinge caused a significant increase in displacement (1.7 mm vs 2.35 mm in ER), which was reduced by an additional plate (0.4 mm vs 0.6 mm in ER and IR). These differences may come from the testing setup (knees were tested with an axial load in 45° of knee flexion) and the 4.2-mm plate. The aforementioned studies did not test biplanar osteotomies, which are considered the standard, as they have been found to have an improved bone healing potential compared with the monoplanar osteotomies.^37^ This may be due to the increased bone contact area^4,38^ and the reduced rotational displacement, as the metaphyseal segment rotates against the protruding bone of the biplanar cut.

The results of the present study are of clinical relevance, considering that nonunions of the osteotomy gap possibly caused by hinge fractures remain the key concern in DFOs.^33^ In a recent clinical study, Rupp et al^31^ identified dislocated hinge fractures >2 mm as a critical threshold value for lateral closing wedge DFO malunion. The retrospective analysis of 79 patients revealed medial hinge fractures in 48% of cases, demonstrating a higher risk of malunion than those with intact medial cortical bone (13% vs 2%). Even though this threshold was derived from a lateral closing wedge osteotomy, it may also apply to the tested LOW-DFO and MCW-DFO in the present study. This finding suggests that hinge fractures may impair bone healing of the distal femur due to increased rotational displacement across the osteotomy gap. Although the plate fixation did not completely restore the torsional stiffness of the bone-implant construct, the rotational displacements could be restored to the same level as that of the nonhinge fractured bone so that an additional plate fixation may be a solution to prevent nonunions.

The present study had several limitations inherent to the biomechanical testing of cadaveric specimens. First, the biomechanical testing simulated forces acting at time zero when biological factors and osseous integration processes were not considered. Second, an axial load of 400 N and a torsional load of 10 N·m were applied in this study, which were lower than forces acting under full weightbearing during the gait phase. However, the present study was performed to examine displacements of the hinge site and the biomechanical effects of an additional fixation using a predestructive loading level and simulate early postoperative rehabilitation. These loads were chosen to ensure consistency with the current literature; nonetheless, it should be noted that higher axial loads could have altered the results. Third, the used cadaveric specimens were older than the patients normally treated with a DFO; thus, the stiffness and displacement may be underestimated or overestimated because of the lower bone density. However, each specimen was serially tested so that the material properties within each specimen were constant for each testing condition. Fourth, the present study only investigated the displacement at the hinge site under axial compression and torsional loads. Other loading conditions, which may also impair bone healing, were not included.^17,19^ Last, the provided data only refer to type 1 hinge fractures (along the osteotomy plane) after MCW-DFO and LOW-DFO and may not be directly transferred to other hinge fractures.^29,39^

## Conclusion

Hinge fractures after MCW-DFO and LOW-DFO caused decreased rotational stiffness of the bone-implant construct and increased displacement at the fracture site. In contrast, the axial stiffness remained unchanged in our cadaveric model. When considering an osteosynthesis of a hinge fracture in a DFO, an additional plate fixation was the construct with the highest stiffness and least displacement, which could restore intact hinge rotational displacement.
